# Perspectives of EFL learners and teachers on self-efficacy and academic achievement: The role of gender, culture and learning environment

**DOI:** 10.3389/fpsyg.2022.996736

**Published:** 2022-10-20

**Authors:** Liyuan Liu, Muhammad Amir Saeed, Nasser Said Gomaa Abdelrasheed, Goodarz Shakibaei, Ayman Farid Khafaga

**Affiliations:** ^1^School of Foreign Languages and Cultures, Southwest Jiaotong University, Chengdu, China; ^2^Department of English Language and Literature, College of Arts and Applied Sciences, Dhofar University, Salalah, Oman; ^3^School of Educational Studies, Universiti Sains Malaysia, George Town, Malaysia; ^4^Department of Education, College of Arts and Applied Sciences, Dhofar University, Salalah, Oman; ^5^Department of Mental Health, College of Education, Minia University, Minya, Egypt; ^6^Department of English, Islamic Azad University of Ahvaz, Ahvaz, Iran; ^7^Department of English, College of Science and Humanities, Prince Sattam Bin Abdulaziz University, Al-Kharj, Saudi Arabia; ^8^Department of English, Faculty of Arts and Humanities, Suez Canal University, Ismailia, Egypt

**Keywords:** co-education, mixed-gender classrooms, self-efficacy, academic achievement, sociocultural context, learner's perspectives, teachers' perspective, self-confidence

## Abstract

The Omani socio-cultural context, the mono-gender educational system in schools, and the learning environment at the higher educational institutions significantly affect learners' self-efficacy and academic achievement in the mixed-gender EFL classroom. Different studies have revealed both positive and negative implications of mixed-gender classrooms, especially for those who came from a mono-gender learning environment. The adjustment phase for the tertiary learners from school to the university is not only crucial but also significant for the continuation of higher education. The effects of socio-cultural factors on self-efficacy and academic achievement have not been studied in depth, particularly in eastern countries. So, the current study aimed at investigating the role of gender, learning background, socio-cultural circumstances, and the effect of the learning environment on EFL learners' self-efficacy and their academic achievement within the scenario of the prevailing culture and traditions in the Dhofar Region. To conduct this study, mixed research methods (qualitative and quantitative) have been adopted to investigate the perceptions of both teachers and learners. The sample of the study consists of 117 EFL learners ranging between 18–22 years of age and 25 EFL teachers ranging between 35–60 years of age. We used separate surveys for students and the teachers and interviewed students and teachers on a random basis. The results demonstrate that both genders were comfortable in segregated classes. The results also reveal that female learners were active learners and better performers than male learners in the school learning environment. The students reported that social restrictions discouraged them from mixing with the opposite gender in classroom activities and oral discussions. Most teachers believe that, compared to male learners, female learners performed better and were more engaged and responsive to different learning situations. The study found that there were statistically significant differences between both genders in terms of the effects of socio-cultural environment, self-efficacy, and the learning environment. Female learners were better than male learners in mono-gender schools, and they have higher self-efficacy than male students at the university. In conclusion, EFL teachers should consider the socio-cultural context, learners' learning background, and other challenges of learners to bring out positive outcomes in a mixed-gender classroom.

## Introduction

Transitioning from mono-gender classrooms to mixed-gender classes at the university is a critical period for learners (Fokkens-Bruinsma et al., 2021), especially for those learners who have never been in a co-education learning environment. Mixed-gender classes have been endorsed and appreciated by educators and society in the last few decades. The mixed-gender classroom is the place where both males and females study together under the same learning conditions and the same setting. According to Evans (2014), mixed-gender classes help learners understand the similarities and attitudes of the opposite gender in a classroom during the period of formal education. Though gender-segregated classrooms were common in the 19^th^ century in the West, Europe, and Russia to instill self-efficacy and management skills (Bajaj, 2009), the model of the mixed-gender classroom was initiated in the West to showcase gender equality in education (Hussain, 2020). Mixed-gender classes provide learning opportunities for both genders under similar learning conditions and in the same learning environment. Educational institutions where a mixed-gender learning environment is provided help both boys and girls in building social maturity and developing communication skills (Ahmad et al., 2014; Almasri et al., 2021).

Though there is much debate about the effectiveness of mixed-gender and mono-gender learning environments, research in recent years has shown that mixed-gender classrooms are not very productive and do not produce the expected results (Hussain, 2020). However, it is unanimously agreed that both genders have specific personality traits and characteristics that differentiate them marginally. They have different learning patterns and self-efficacy that affect academic achievement. Self-efficacy minimizes the disparity between school and university learning environments and academic success at a higher educational institution (Zysberg and Schwabsky, 2021). Self-efficacy is an area that has focused on girls and boys in the different ways in which it affects their academic performance and their overall well-being and success in life (Namaziandost and Çakmak, 2020). It is found that in a mixed-gender classroom, there is a vast difference in self-efficacy of both genders compared to single-gender classrooms (Li and Singh, 2021). Self-efficacy has recently gained growing attention as one of the strong influences that affect student academic achievement (Nasir and Iqbal, 2019). High achievers have higher self-efficacy compared to low achievers (Yasin et al., 2020), and the learning environment and socio-cultural background contribute to developing self-efficacy in learners (Harvey et al., 2017; Zysberg and Schwabsky, 2021). The academic achievement of EFL learners is directly related to their type of learning environment, parents' education, and socio-economic situation (Hussain, 2020). There is abundant evidence that confirms that self-efficacy has a positive effect on academic success and other related academic activities (Lee et al., 2014).

Aragonés-González et al. (2020) argued that the classroom environment, coursebooks, gender, and cultural setting shape learners' attitudes toward learning. Gender roles can be controlled and readjusted by modifying teaching practices and teaching materials. Their study revealed that the education system needs to be reformed to give an equal representation of both genders in the curriculum and teaching resources. They found that socio-cultural challenges are the barriers to girls' education and affect the school dropout rate. They further emphasized the necessity of teacher training to understand gender differences and requested governmental intervention to reform the course materials.

Park (2018) reported that in a Korean context, co-education classrooms do not have a significant effect on academic achievement for both genders. However, the girls with low motivation and caliber were anxious in mixed-gender classes and it negatively affected their academic achievement. He also confirmed that the girls are high achievers compared to the opposite gender in a mixed-gender classroom (Lavy and Schlosser, 2011; Al Murshidi, 2014; Alhazmi and Nyland, 2015; Eisenkopf et al., 2015). Park (2018) observed that mixed gender classes have a positive effect on female learners' choice of course selection, particularly when he observed a significant increase in their selection of science courses compared to male learners. He also highlighted the fact that the selection of science and engineering courses on the part of male learners is due to the high salary paid to scientists and engineers as compared to other professions.

It has also been observed that classes with a larger proportion of girls have a positive effect on classroom discipline, teacher-student relationships, and the learning environment (Lavy and Schlosser, 2011). To date, due to the absence of appropriate data, the gender composition of peer groups has received little attention. Eisenkopf et al. (2015) observed empirically sound evidence in their study that a single-gender learning environment has a positive effect on females studying science courses in Switzerland. The educational environment has a direct effect on students' attitudes toward learning, self-confidence, and ultimately affects students' achievement in a gender-segregated classroom. In the future, a study on the impact of the female-dominated classroom on male students' academic achievement is recommended.

The modernization of education across the world coupled with an emphasis on promoting the English language has altered the landscape of higher education in the West and Europe. In Middle Eastern societies, educators have started revisiting the effectiveness of co-education in improving the academic standards of students of both genders. Mathew et al. (2013) and Coskun (2014) observed that females in the Arab co-educational context demonstrated anxiety and discomfort in learning and class participation. However, this anxiety increased their productivity, and they showed better performance academically compared to their male counterparts.

In the UAE, in mixed-gender classrooms, female learners experienced cultural barriers to communicating with their male counterparts freely. Male students' low performance is the result of a lack of motivation and low self-efficacy in a co-educational learning environment (Al Murshidi, 2014). The school learning environment and learners' self-efficacy, which are interrelated (Machell et al., 2016), also affect both genders in the Arab co-educational context. In the Saudi context, gender-segregated classrooms have been a subject of discussion by local researchers in terms of striking a balance between the modernization of learning, local culture, and religious point of view (Alhazmi and Nyland, 2015).

The government in Oman aims at developing global citizenship in the students studying at higher education institutions (Al-Maamari, 2014), and it has spent heavily on higher education since the renaissance in 1970 (Al-Mahrooqi and Denman, 2018). Owing to the dominating culture in Oman, both genders in a co-educational environment are bound to stay away from mixed-gender mingling and maintain physical distance, avoid cross-gender communication during class activities, and have different learning styles (MacKenzie, 2016). Within such a contextual learning environment, learners encounter certain socio-cultural issues, since both genders have different learning styles and learning backgrounds (single-gendered schools; co-educational schools, international schools). Therefore, learners exhibit differences in their self-efficacy, self-confidence, and motivation in learning. Researchers in the context of Oman discovered that high school graduates and students enrolled in foundation year programs in Oman have a lack of self-confidence, low proficiency in the foreign language, a lack of interest in reading and domestic responsibilities, and they face specific learning challenges such as mixed-gender classes, the difference between school and university learning environments, anxiety in learning, and cultural barriers while studying at university (Al-Issa and Al-Bulushi, 2012; Al-Mahrooqi and Denman, 2018).

Therefore, this study aims to identify the extent to which co-education helps students not only in learning their subjects but also in developing their personality and building their confidence (self-efficacy). Students who graduate from single-gendered schools have a low level of English and, hence, they are required to do a Foundation Year (Al-Mahrooqi and Denman, 2018). The students who enroll in colleges and Dhofar University come from complex cultural backgrounds — Bedouins and fishermen communities, along with the students residing in the city of Salalah. The overwhelming culture is a mixture of the regional variations that restrain the mingling of males and females. Most of the schools in the Dhofar region are gender-based schools, except for some private schools that have adopted mixed-gender classes (Risse, 2019; Watson et al., 2019). When students from these regions and cultural backgrounds enroll in colleges and universities, they not only find male and female students studying together but also are taught by people from different genders and nationalities. This poses a real challenge to a majority of students in coping with their educational aspirations. Crucially, low language proficiency, combined with cultural shock, creates a sense of otherness in students who attend co-educational colleges and universities, which contributes to digression and low self-efficacy. “Though culture shock is expected every time someone changes one's cultural environment, the impact varies from person to person and the experience can alter one's perception of the anticipated outcome” (Al Murshidi, 2014, p. 100). Research has shown that there is a link between gender and learning styles (Kaiser, 2006; Glynn et al., 2007; Ning et al., 2010; Nazir et al., 2018). The situation is no different in the area in which the researchers have done the study.

Having reviewed the existing literature, there is a dearth of comprehensive studies that discuss the relationship between culture, learning environment, and gender and its effect on learners' self-efficacy and academic success in a mixed-gender classroom in an Omani context. Therefore, the current study has been conducted to determine the role of gender, culture, and learning environment on learners' self-efficacy and achievement in a mixed-gender classroom in the context of a private university in Oman. The current study tries to bridge the gap in the existing literature in the context of Oman by highlighting the role and significance of gender, learning environment, and culture on the learners' learning process in a mixed-gender classroom where culture, gender differentiation, and previous learning environment are the dominating factors that affect and shape learners' self-efficacy and confidence in learning and their academic success. This study is anticipated to contribute to other higher educational institutions that have similar learning environments and learners' self-efficacy differentiation.

## Literature review

### Mixed-gender classroom vs. single-gender classroom

In both gender-segregated and mixed-gender classrooms, teachers tend to have a consistent teaching methodology and gender does not have any kind of effect on their teaching approaches. Teachers perceive girls as high achievers compared to boys. Both parents and teachers observe that gender-segregated classrooms have a positive effect on building social skills in both learners. Academic performance in mathematics by both genders is notably better than in a coeducational learning environment. When compared to mixed-gender classes, many teachers found homogeneous-gender classes to be more intensive and engaging (Becker, 2013).

According to Evans (2014), single-sex schools in Zambia are considered healthy spaces for personal development and free of male dominance compared to mixed-gender schools, which are considered places of unfair power dynamics. Certain findings suggest that single-sex schools contribute to higher academic performance, self-efficacy, self-concept, and learner's autonomy (Hussain, 2020). Girls studied in the mono-gender learning environment in Zambia show fear about their encounters with boys in a mixed-gender classroom. Females who studied in single-gendered classrooms faced various challenges in transitioning to higher education and jobs with males. They felt less confident and had low self-efficacy, which affected their performance negatively in the class (Evans, 2014).

For Yalcinkaya and Ulu (2012), the students in Kazakhstan concentrated better in segregated classrooms; however, in mixed-gender classrooms, most of the students were distracted by the presence of the opposite gender. The mixed-gender classroom provides a competitive and interactive learning environment for the students. Most of the students felt that the segregated learning environment was positive in terms of productivity and learning. In some situations, the involvement of the opposite gender in classroom discussion harmed students' efficacy and motivation in learning.

### Mixed-gender classroom and academic achievement

A great deal of research has been carried out to understand the variables affecting academic achievement. However, only a few studies have focused on the role of gender in predicting a student's academic success (Whipple and Dimitrova-Grajzl, 2021). Female students not only surpassed their male counterparts in academic achievement but also in classroom activities and participation. Significantly, the lack of motivation pertaining to male learners is not the reason for their low academic performance; however, their low self-efficacy and lack of confidence in front of the opposite gender cause an academic weakness to emerge. Female students' anxiety had a positive effect on their performance and academic achievement. However, it needs to be explored further in the socio-cultural context (Mathew et al., 2013; Fallan and Opstad, 2016). According to Whipple and Dimitrova-Grajzl (2021), gender has a significant role in academic achievement. They maintain that male learners achieve higher grades compared to female learners, who have a moderate effect on their learning in the presence of the opposite gender.

Hussain (2020) found that learners from a single-gender learning environment in Pakistan scored higher on an English Language proficiency test. The social and gender environments in single-gender schools can be related to this disparity in results. Students' lower scores in co-educational schools indicate that they are distracted by the presence of members from the opposite gender and, as a result, they achieve lower grades in a co-educational environment compared to segregated schools. In the Indian context, Harinarayanan and Pazhanivelu (2018) observed that the demographics of learners, school learning environment and educational facilities, and students' mono-gender and mixed-gender learning backgrounds in school have a significant effect on learners' academic achievement. In his study, Becker (2013) focused on the gender of the teacher and reported that the teacher's gender does not affect learners' academic achievement and engagement in the classroom.

### Mixed-gender classroom and culture

The cultural aspect has always been one of the dominating factors in shaping a learner's personality and attitude toward learning in the Persian Gulf region. Higher education in the Gulf region is a relatively new phenomenon with a predominant cultural aspect that is completely different from the Western educational model. This cultural dominance has led some of the higher educational institutions in the Gulf to have segregated campuses for both genders, especially in the UAE and Saudi Arabia (Parahoo et al., 2013; Song, 2019).

In Saudi Arabia, there is a clear cultural division of both genders in terms of domestic affairs, business, education, and social activities. Females in the Saudi context have to study in gender-segregated institutions and have to readjust themselves to linguistic, cultural, and religious challenges to progress in education, since the majority of females are responsible for domestic chores (Alhazmi and Nyland, 2015; Song, 2019). Learners' self-efficacy, motivation, socialization, gender roles, and EFL learning ability are shaped by the cultural norms and learning environment provided by society (Song, 2019). In an Omani context, both genders have a lack of motivation and self-efficacy in learning a foreign language (Al-Mahrooqi, 2012).

### Mixed-gender classroom and self-efficacy

Self-efficacy is the learner's belief or perception of one's learning capability, self-efficacy, and learning perception. However, there is very little research about the relationship between learning, self-efficacy, and academic achievement during the transition from high school to university (Van Herpen et al., 2017). In a mixed-gender classroom, both genders exhibit an equal level of satisfaction in learning, and teachers' learning background is not a significant factor that affects students' motivation and inclination toward learning (Harvey et al., 2017). Learners who have high self-efficacy and are motivated to participate in classroom activities actively compared to learners with a lack of confidence and low self-efficacy (Becker, 2013). In Kenya, females who are studying English as a Foreign Language demonstrate high self-efficacy compared to males (Makini et al., 2020).

The Gulf region has its own specificities in terms of a relatively new higher education sector and a different socio-cultural setting as compared to the western empirical contexts of most studies undertaken (Becker, 2013). Kuwaiti females are more anxious and less confident in front of males. In certain situations, it is also observed that senior students are more confident and autonomous compared to tertiary learners. Omani male students are more anxious and shier in front of female students, and feel insecure and sensitive for being mocked by their female counterparts. Similarly, in certain classroom activities and situations, female learners have exhibited a similar kind of sensitivity and anxiety as is the case for male learners (Mathew et al., 2013).

### Mixed-gender classrooms and learning environment

The academic environment affects both learning and learners' behavior in a specific way in an academic setting. However, the learner's demographic has a negligible effect on the learner's learning environment. It is found that the academic environment has a positive effect and relationship with the individual's academic achievement (Benbenishty et al., 2016). However, it is not significantly related to increasing self-confidence in learners (Rocconi et al., 2020). In the Gulf context, the majority of the higher educational institutions are heavily supplied with the latest teaching and technological equipment, which have transformed communication and interaction with the students. This highly tech-loaded environment, with the availability of Virtual Learning Management Systems, email, online forums, and blogs, has transformed learning. Also, it has been discovered that students expect to be connected with their instructors and classmates *via* various internet and high-tech resources (Parahoo et al., 2013).

According to Harvey et al. (2017), a learning environment in a gender-mixed classroom is associated with cultural obligations and social norms that are not necessarily characterized by genders. A study by Fortin et al. (2015) reveals that race, living standard, and family background of learners shape a learning environment in a mixed-gender classroom in schools. In most situations, these social circumstances are advantageous to male learners as compared to female ones. In their study, Makini et al. (2020) argue that teachers can also contribute to the learning environment by understanding the personality traits of both genders and by adapting their curriculum and teaching materials to these variables. In an Indian context, female learners have a better image of the learning environment than male students, and they take more advantage of educational resources (e.g., libraries and laboratories) and educational activities in comparison to boys (Harinarayanan and Pazhanivelu, 2018).

The theoretical framework of this research is based on two aspects of Bandura and Bussey's Social Cognitive Theory of Cultural Context and Gender Development and Differentiation (Bussey and Bandura, 1999). According to the Social Cognitive Theory of Gender Development, gender conceptions are developed through the mechanisms of motivation, self-regulation, and complex experiments linked with gender self-conduct. Gender development is characterized by a socio-cultural setting where people gain opportunities and face particular challenges based on their beliefs and self-conceptions, their professional pathways, and societal stereotypes about genders. Furthermore, changes in technology and sociocultural situations influence gender behavior (Bussey and Bandura, 1999). “People contribute to their self-development and bring about social changes that define and structure gender relationships through their agentic actions within the interrelated systems of influence” (Bussey and Bandura, 1999, p. 676).

## Current study

Most of the studies on mixed-gender classrooms and segregated classrooms have been conducted in Western countries (Hussain, 2020), and the investigation on the effect of mixed-gender classrooms of students' academic achievement, self-efficacy, and learning environment in higher educational institutions in the Persian Gulf region has been an unexplored area (Parahoo et al., 2013), specifically in Oman. Therefore, this study intends to investigate whether mixed-gender classes are effective in bringing about a conducive learning and competitive environment for both genders within the scenario of the prevailing culture and traditions in the Dhofar Region. The primary objective of this study is to investigate the effectiveness of co-education for both genders concerning culture, self-efficacy, academic achievement, and a positive learning environment. The focus of this research is also to probe the attitudes of both genders who studied in a single-gendered learning environment in schools. A mixed-method approach based on both quantitative and qualitative methods was adopted in this study.

## Research questions

1. What are the perspectives of EFL learners on self-efficacy and academic achievement in a mixed-gender classroom?
2. What are the perspectives of EFL teachers on learners' self-efficacy and academic achievement in a mixed-gender classroom?
3. What is the effect of culture and the learning environment on learners' academic achievement?
4. What is the effect of culture and the learning environment on learners' self-efficacy?
5. Do the perspectives of EFL students on the school environment, university environment, and teaching and learning environment in the university differ according to gender?

## Methodology

### Study design

The study followed a mixed approach whereby researchers collected and analyzed both quantitative and qualitative data within the same study (Creswell and Plano-Clark, 2011). We distributed the link to questionnaire to the students with open-ended questions as well as closed-ended questions in the classrooms after obtaining the permission of the course teachers, and this was done through the first researcher. The link to the questionnaire for teachers with open-ended questions as well as closed-ended questions was also distributed to their offices after obtaining their consent. Some students and teachers were selected randomly for interviews to recognize their perspectives on the impact of the mixed-gender environment on their self-efficacy.

### Participants

The sample of the study consists of 117 university students from different regions of the Sultanate of Oman: 32 males (27.4%) and 85 females (72.6%). In terms of age ranges, the participants ranged between 18 years and 21 years, with mean age *M* = 19.25 (SD = 1.32). A total of 31.6% of the students are studying in the foundation program; 27.4% are studying in the first year; 24.8% are studying in 2nd year; and 7.7% are studying in the 3rd year. The participants were recruited from different courses at Dhofar University and were randomly selected.

Twenty-five language teachers were included in the current study. Their ages ranged between 35 and 55, with a mean age of 42.56 (SD = 3.72); Seven with a master's degree and 18 with a Ph.D. degree. Most of the teachers were assistant professors (18, 72%) with about 5–10 years' experience of working at Dhofar University, and they were from diverse cultures and different nationalities. Twelve teachers (48% of them) were from the Foundation Program, and 13 (52%) were from the College of Arts and Applied Sciences.

### Data collection

The study employed a cross-sectional research design. Before commencing the data collection phase, ethical approval was obtained from the research department at Dhofar University to conduct the study on university students. Data were collected from students in several courses at Dhofar University. Data were collected from 12 March 2020 to 21 April 2020 during the Spring semester of the academic year 2019/2020. A letter of invitation, a consent form, and the instrument package were given electronically to the participants. The study was approved by the ethics committee of the author's university. The interviews were conducted *via* Zoom sessions from April 25^th^ to May 10^th^, 2020. A zoom link was sent to students and teachers who were selected for the interviews. The process of data collection followed the voluntary principle. All the subjects provided written informed consent per the Declaration of Helsinki. However, they were free to withdraw during the research process, and their privacy and personal information were kept confidential.

### Measures

The online survey was developed by the authors and information was gathered from two different informant perspectives (i.e., teachers and learners).

#### Students' survey

For the student survey, the first part includes sociodemographic variables like gender, age, level of study, type of school (single or mixed gender), college, and region. The second part of the survey items consists of three sub-dimensions. The first sub-dimension is about the previous school environment including seven items like “I performed well when I was in school,” “I never felt shy to participate in class activities in the school.” The second sub-dimension is about the socio-cultural environment, including eight items like “my culture does not allow me to study in a mixed-gender class,” “social restrictions in my society discourage mixing with the other gender.” The third sub-dimension is about learners' efficacy and the teaching and learning environment at the university including 15 items like “I feel depressed if I fail to answer in front of the other gender,” and “mixed-gender class environment encourages competition among all the students.” The survey was designed on a Likert scale with four choices, namely, (1) Strongly disagree, (2) Disagree, (3) Agree, and (4) Strongly agree. After completing the construction of the questionnaire, the content validity was verified by presenting it to five reviewers specializing in psychology and education, to ask their views on the questionnaire statements and their relevance to students, and their relevance to what was set to be measured. The scale was modified based on their opinions. Cronbach's alpha was calculated, and it was 0.69 and it is an acceptable reliability coefficient.

#### Teachers' survey

For the teacher's survey, the first part includes sociodemographic variables like gender, age, experience, nationality, qualification, college, and academic rank. The second part of the survey deals with the perspectives of teachers on the learners' self-efficacy and academic achievement in the mixed-gender classroom. It consists of 17 question items like “I encourage both genders to participate equally,” and “female learners are more active than boys during the lecture.” The survey was designed on a Likert scale with four choices, namely (1) Strongly disagree, (2). Disagree, (3) Agree, and (4) Strongly agree. After completing the construction of the questionnaire, the content validity was verified by presenting it to five reviewers specialized in psychology and education, to ask their views on the questionnaire statements and their relevance to students, and their relevance to what was set to be measured. The scale was modified based on their opinions. Cronbach's alpha was calculated, and it was 0.73 and it is an acceptable reliability coefficient.

#### The interview questions

Some students and teachers were randomly chosen for the interview. The interview questions consist of three questions for the students, such as “what's the role of culture in developing self-confidence?” and six questions for the teachers, such as “how can both the genders be motivated to perform confidently in the classroom?” and “in your views, how can teachers create an environment of competition and learning among both genders?”

## Data analysis

The data were analyzed using the SPSS program (Version 26), and the interview questions were manually analyzed to extract the main themes from the teachers' and students' responses. Missing data were imputed when necessary in a random manner. Only a very small proportion of the data were missing (<2% overall). The normality distribution of the data was verified before the analysis by calculating the skewness and kurtosis coefficient, and their values were very close to zero (0.213 and 0.367). This indicates that the data distribution was close to the normal distribution. Outliers were also examined, and no outliers were found, so, all participants' responses were considered when analyzing the data. We used many statistical techniques including means, standard deviations, correlation, *T*-test, and Cohen's *d*.

## Results

Regarding the research questions No. 1, 3, and 4, we analyzed the responses of the students by using mean and standard deviation. The results are given in the Table 1.

**Table 1:** Responses of the study sample to the areas of the questionnaire in all its items.

**Area**	**S. No**.	**Statement**	**Mean**	**SD**	**Percentage**	**Agreeable level**
**School environment**	1	I performed well in my studies when I was in school	3.93	1.09	78.60	High
	2	I participated actively in school activities	3.57	1.40	71.40	High
	3	I never felt shy to answer teachers' questions in school	3.72	1.24	74.40	High
	4	I was competitive in school	3.79	1.36	75.80	High
	5	I was comfortable in a single-gender learning environment	4.21	1.22	84.20	Very1 high
	6	I scored good grades in school	3.91	1.11	78.20	High
	7	I enjoyed working in a group	4.03	1.20	80.60	High
**The average of the first field (school environment)**			3.92	0.73		High
**Socio-cultural environment**	8	I was shocked to see boys and girls studying in the same class	3.10	1.47	62.00	Medium
	9	My culture does not allow me to study in a mixed-gender class	2.94	1.33	58.80	Medium
	10	There are more boys in my class	2.98	1.47	59.60	Medium
	11	In my culture, girls are not allowed to speak more in front of the boys	3.87	1.40	77.40	High
	12	Some of my cultural rituals and social norms do not provide an opportunity to study at home	3.00	1.44	60.00	Medium
	13	Social restrictions in my society discourage mixing with the other gender	3.47	1.34	69.40	High
	14	My culture prefers separate classes for gender	3.33	1.54	66.60	Medium
	15	I prefer to study in a single-gender class	3.46	1.52	69.20	High
**The average of the first field (University and Omani culture)**			3.25	0.82		Medium
**Self-efficacy and teaching**	16	I believe I can achieve the goals that I have set for myself	2.83	1.46	56.60	Low
**and learning environment in** **university**	17	In general, I believe I can score higher grades in my assessments in a mixed-gender classroom	2.29	1.44	45.80	Low
	18	When facing a difficult task, I am certain that I will accomplish it	3.79	1.38	75.80	High
	19	I feel depressed if I fail to answer in front of the opposite gender	3.75	1.41	75.00	High
	20	I can successfully overcome many learning challenges in a mixed-gender classroom	3.64	1.32	72.80	High
	21	I am confident that I can participate efficiently in many classroom activities	3.38	1.49	67.60	Medium
	22	Compared to the opposite gender, I am confident that I can do most tasks efficiently	2.83	1.57	56.60	Medium
	23	I learn better in a mono-gender classroom	3.89	1.50	77.80	High
	24	A mixed-gender class environment encourages competition among all the students	3.88	1.37	77.60	High
	25	I like to be recognized as the best student among both genders	3.35	1.36	67.00	Medium
	26	I get enough opportunity to participate in the mixed-gender class	2.89	1.38	57.80	Medium
	27	The teacher provides an opportunity for both genders to participate in the class equally	3.06	1.46	61.20	Medium
	28	There is enough interaction between boys and girls in-class activities	2.54	1.39	50.80	Low
	29	The classroom activities at university help me improve my knowledge.	3.31	1.51	66.20	Medium
	30	The university learning environment helps me to be competitive in my studies	3.67	1.21	73.40	High
**The average of the first field (University and Omani culture)**			3.34	0.58	66.80	Medium
***The Average of the questionnaire as a whole***			3.46	0.48	69.20	High

### School environment

The results demonstrate that both genders were comfortable in segregated classes. For example, “I was comfortable in a single-gender learning environment” the student's approval was very high on this item, i.e., 4.21/5.00, which led them to participate actively in the classroom activities, group work, and competitive studies, and ultimately resulting in achieving high scores in school. For example, most students reported that “I enjoyed working in a group,” “I performed well in my studies when I was in school.” with an average of 3.93/5.00.

### Socio-cultural environment

According to the results, the students reported that the social restrictions discourage them from mixing with the opposite gender in classroom activities and oral discussions. For example, “In my culture, girls are not allowed to speak more in front of the boys” with an average acceptance of 3.87/5.00. Both genders also affirm that though their culture does not restrict mixed-gender classes, most of the students prefer to study in a segregated classroom. For example, “I prefer to study in a single-gender class” was approved by 3.46/5.00. Many tertiary learners are even shocked to see gender-mixed classes as an initial experience in the university, which also impacts learners' efficacy and motivation. Consequently, the students realize that the socio-cultural environment not only affects their self-efficacy and motivation but also has a negative effect on their academic achievement.

### Self-efficacy and university teaching and learning environment

According to the average of the results, the students have a medium level of self-efficacy that is 3.34/5.00. Both genders are aware of the fact that mixed-gender classrooms provide them with a competitive learning environment. For example, “A mixed-gender class environment encourages competition among all the students,” with an average of acceptance of 3.88/5.00. However, both genders emphasize that they have high self-efficacy in mono-gender classrooms. For example, “I learn better in a mono-gender classroom,” with an average of 3.89/5.00. As a result, there is very limited/no interaction between both the genders during the classroom activities. For example, “There is enough interaction between boys and girls in-class activities,” with an average of 2.54/5.00. Also, students feel uncomfortable if they are unable to respond successfully in the presence of the other gender. For example, “I feel depressed if I fail to answer in front of the opposite gender,” with an average of 3.75/5.00. Ultimately, both genders believe that they will not be able to achieve high scores in the assessments, such as “I believe I can score higher grades in my assessments in a mixed-gender classroom.” with an average of 2.29/5.00.

Both genders acknowledge that the university teaching and learning environment provides them with a better and more competitive learning environment, for example, “The university learning environment helps me to be competitive in my studies.” with an average of 3.67/5.00. The learners find that the learning environment at the university is conducive to improving their knowledge. For example, “The classroom activities at university help me improve my knowledge.” The learners agree that the teachers are very supportive to both genders learning at the university. For example, “The teacher provides an opportunity to both genders to participate in the class equally,” with an average of 3.06/5.00.

Interviews with students were also analyzed, and these interviews showed that the transition from education in a same-gender school to university education alongside the opposite gender represents an obstacle to their academic achievement; that this transition has negatively affected their participation in class in front of the opposite gender; and that many of them prefer not to speak in front of the opposite gender because they feel ashamed and embarrassed.

“*Sometimes, I cannot participate in the class because of some boys or girls.”*

“*Yes, because boys are in the class.”*

“*Yes, the presence of boys.”*

“*Yes, there are many girls in my class.”*

Regarding the results of research question No. 2, we analyzed the responses of the teachers by using mean and standard deviation. The results are given in the table below.

The results displayed in Table 2 indicate that most teachers do not have anxiety or obligation to teach mixed-gender classes. Moreover, they encourage both males and females to participate equally. Most teachers believe that, compared to male learners, female learners perform better and are more engaged and responsive to different learning situations. The teachers reported that girls are more responsible for completing home tasks and assignments and achieve higher grades compared to the opposite gender. However, some of the language teachers have a different opinion. Teachers were divided on the positive effect of mixed-gender classes and their effect on academic achievement.

**Table 2:** Responses of teachers on the teachers' questionnaire.

**S. No**.	**Statement**	**Mean**	**SD**	**Percentage**	**Agreeable level**
1	I feel comfortable teaching in mixed gender classes	4.33	2.10	86.60	Very high
2	I encourage both boys and girls to participate equally	4.15	0.87	83.00	High
3	In my class girls do better than boys	4.41	1.54	88.20	Very high
4	The majority of students in my class are boys	2.75	0.98	55.00	Medium
5	Girls are more responsive in my class	4.06	2.01	81.20	High
6	Most of the girls in my class do their homework regularly	3.98	1.42	79.60	High
7	Girls score better than boys in my class	4.22	1.34	84.40	Very high
8	Mixed gender classes encourage competitive spirit	2.65	0.76	53.00	Medium
9	Students' performance improves in mixed gender class	2.68	1.11	53.60	Medium
10	I prefer gender segregated classes	2.47	0.65	49.40	low
11	Mixed gender classes discourage students' active participation in class	3.87	1.22	77.40	High
12	Student communication is either limited or controlled in my class	4.31	1.28	86.20	Very high
13	Mixed classes have little or no effect in building confidence in both the genders	3.66	1.43	73.20	High
14	Boys participate actively than girls in my class	2.12	0.81	42.40	low
15	Girls are more regular in attending the classes than boys	4.03	1.08	80.60	High
16	Both the genders spend most of the class time passively	2.77	1.14	55.40	Medium
17	Students native culture does not allow mixing of the two genders	4.26	0.35	85.20	Very high

Nearly all teachers opt for gender-mixed classes compared to gender-separate classes. Half of the teachers declined the idea that co-education has a negative effect on the classroom learning environment and students' communication with the teacher. However, the other 50% reported that the combined classes lead to limited or no communication between the opposite genders for socio-cultural reasons. Most teachers believe that despite the challenges, students are motivated and have self-efficacy for learning.

Regarding class participation, the teachers reported that both genders are active in learning in the classroom and that none of the genders is passive in the presence of the opposite gender. Evidently, the teachers observed that the female learners were more vigilant and punctual in attending lectures. Lastly, all the teachers agreed that the students have socio-cultural restrictions which prohibit them from mixing freely with the opposite gender in the class during different classroom activities.

Interviews with teachers were also analyzed, and these interviews showed that many students lack confidence in mixed-gender classes. Most of them agreed that the reason for the lack of confidence, and the unwillingness to speak in the classroom is due to the presence of the opposite gender.

“Many students lack confidence for many different reasons. One important reason is they feel uncomfortable in front of students of the other gender. (USA)”

“Yes, they often complain that in mixed classes they don't feel comfortable expressing themselves. However, I believe in most cases this could be used as an excuse not to speak in English and/or their lack of commitment. (Italian)”

“It is true that students lack confidence. I believe the mixed class is a means to build confidence and overcome their fear, shyness, and inhibitions. (Indian)”

Regarding the results of the research question No. 5, we analyzed the responses of the students by using a *T*-test after checking the terms of its use. The results are given in the table below.

It is evident from Table 3 that there are statistically significant differences at the level of 0.01 between male and female students of Dhofar University regarding effective participation in the school environment, the effect of socio-cultural environment, and self-efficacy. The differences were in favor of female students, which means that female students were more actively participating in and completing both in- and out-of-class activities than male students. Moreover, there are statistically significant differences between both genders in terms of the effect of the socio-cultural environment on their learning at university. This confirms that the mixed-gender learning environment has a negative impact on female students, who were better than male students in single-gender schools, while positively affecting female students in terms of classroom participation and interacting in mixed classes at the university. There were significant differences in the self-efficacy of male and female EFL learners. Evidently, female learners have higher self-efficacy than male students at university.

**Table 3:** The significance of the differences between male and female students on the scale dimensions as well as on the total score.

**Dimensions**	**Group**	**Number**	**Mean**	**SD**	***T*-value**	**Sig**.	***d***
School environment	Male	32	3.63	0.82	−2.75	0.01	0.54^**^
	Female	85	4.03	0.66			
Socio-cultural environment	Male	32	3.16	0.75	−2.35	0.01	0.88^**^
	Female	85	3.86	0.83			
Self-efficacy and teaching and learning environment in university	Male	32	3.38	0.67	−1.85	0.05	0.86^**^
	Female	85	3.91	0.55			
Total	Male	32	3.39	0.57	−2.35	0.01	1.05^**^
	Female	85	3.93	0.45			

** Significant at (0.01).

It was evident from the interviews with teachers that female students were more effective in the classroom in the university environment.

“*Girls are more effective in the class than boys. Girls come earlier to the class and have higher scores than boys. (Turkish)”*

“*Girls are more aware of their academic achievement, and they have higher motivation in the class. (Indian)”*

“*Mixed-gender classroom restrict the participation of male students. Male students [show] less participation in the activities and communications in the classroom activities. (USA)”*

Many teachers justify these differences according to the cultural and socio-cultural factors.

“To a certain extent performing or expressing shyness is culturally oriented among both the genders. (Indian)”

“Yes, culture is one of the main obstacles contributing to shyness among both the genders. (Indian)”

## Discussion

After the detailed analysis of the data collected through questionnaires and interview questions, the results indicated that both male and female students were more comfortable and performed better in segregated classes. Moreover, they participated actively in the classroom activities, group work, and competitive studies, which ultimately resulted in achieving high scores in school. In university, the students reported that the social restrictions discourage them from mixing with the opposite gender in classroom activities and oral discussions. Both genders also affirm that their culture does not restrict mixed-gender classes. These results are due to the influence of cultural and social factors of the customs, traditions, and values of the Omani society, which effectively limit the mixing of the genders.

The socio-cultural learning theory, specifically the work of Vygotsky (1986) and Lave and Wenger (1998), accentuate that social interactions are at the heart of learning and cognitive development (Driscoll, 1994). Differences in cultures also incorporate different worldviews. Being aware of such differences helps learners understand and appreciate “different beliefs, behaviors, and values” and interact more effectively with each other (Bennett and Bennett, 2001). This correlates with Kim and Bonk (2002) who employ the notion of culture framework for analyzing cultural differences in the context of cross-cultural learning environments. Yang et al. (2010) also found that we need to be aware of the “blending effect” of cross-cultural learning environments, especially with participants who have the same native language or are from the same geographic region after spending some time closely working together.

EFL teachers found female learners more vigilant, responsive, and engaged in learning compared to male students studying under similar learning conditions. This result is in agreement with the results reported by Harinarayanan and Pazhanivelu (2018), which indicate that female learners have a better image of the learning environment than male students, and they take more advantage of educational resources (libraries and laboratories), as well as educational activities in comparison to male learners. Teachers can also contribute to developing a positive and competitive learning environment to encourage and engage both genders for equal and active participation in learning.

EFL teachers have reported that the majority of female students have high self-efficacy, motivation, readiness for learning, and active participation in academic and extracurricular activities as compared to male learners (Namaziandost and Çakmak, 2020). However, both genders believe that they are confident enough to do different tasks independently and can attain higher grades in mixed-gender classrooms. This study has found out that both genders in a mixed-gender classroom learning environment have demonstrated an adverse effect on their learning, engagement, and academic achievement. Further, the analysis clarified that female learners have been greatly affected in a mixed-gender classroom, as they felt depressed, restricted, and least engaged with the opposite gender. They also felt that the learning environment is less competitive and conducive to learning compared to male learners, who were less affected in a university learning environment. In segregated classrooms and mixed-gender classrooms, Omani students exhibit a significant difference in self-confidence, academic performance, and self-efficacy (Tuzlukova and Ginosyan, 2022). Students with higher grades have demonstrated higher self-efficacy as compared to the average students (Yasin et al., 2020).

The socio-cultural environment has great influence in shaping learners' self-efficacy, motivation, and confidence in learning in the Arab countries (Song, 2019). This study has found that social restrictions and cultural norms have discouraged learners from participating in classroom activities and different oral discussions. Many learners at a tertiary level were even shocked to see the opposite gender in the classroom since most of the learners studied previously in a mono-gender learning environment at school, which negatively affected their self-efficacy, motivation, and academic achievement. Both male and female learners, not only in the Dhofar region but also in the regions of Oman, generally have low self-efficacy and motivation for learning a foreign language (Al-Mahrooqi, 2012; Al-Maamri, 2014; Al-Mahrooqi and Denman, 2018). Omani EFL learners also showed different socio-cultural and emotional challenges while transitioning to the university learning environment (Tuzlukova and Ginosyan, 2022).

Both genders in the school learning environment participated actively in both academic and extra-curricular activities. The analysis demonstrated that female learners were found more vigilant compared to their male counterparts. Also, there is a significant difference for both genders for their active participation in the school and university learning environment. Overall, school environment, socio-cultural setting, and university teaching and learning environment have a significant effect on both genders' self-efficacy and the academic achievement of the students studying at Dhofar University.

The results further showed that there are statistically significant differences at the level of significance of 0.01 between male and female students of Dhofar University regarding effective participation in the school environment. The same holds true for the effect of socio-cultural environment and self-efficacy pertaining to both genders in the Omani EFL setting. That is, female students have higher self-efficacy and academic achievement than male students. These results may be due to the influence of cultural, social, and economic factors. Male students often tend to work to cover the economic costs and refrain from studying, so we often find that female students have higher academic achievement and get higher grades.

These results are in conformity with the findings of Huang's (2013) study, which indicated that female learners displayed higher language arts self-efficacy than males. But it differs from the results of Abdelrasheed et al. (2021), which indicated that between the ages of 16 and 18, males outscore females by the equivalent of 11.6 IQ points, which, in turn, is reflected in their academic achievement. These results also differ from the results reported by Fallan and Opstad's (2016) study, which accentuated the fact that female students have significantly lower self-efficacy level and self-efficacy strength than their male peers.

## Conclusion

The current study aimed to discover the perceptions of EFL learners and teachers on the effect of gender, socio-cultural environment, and the teaching and learning environment on self-efficacy and academic achievement of tertiary EFL learners in a mixed-gender classroom. The effects showed that most of the teachers believe that, in comparison to boys, girls perform better and are more engaged and attentive in a mixed-gender learning environment. The lecturers stated that female learners are more responsive to the assigned tasks, activities, and assignments and obtain better grades compared to their opposite gender. However, some of the language teachers have a different opinion. The EFL teachers reported that both the genders are enthusiastic about learning in the classroom and none of the genders are passive in the presence of the other gender. The study also found that female students are more vigilant and punctual in attending lectures. Almost all of the EFL teachers agree that socio-cultural restrictions restrain them from mixing freely with the opposite gender within the class during classroom activities and discussions.

Additionally, our data demonstrate that both genders were delighted with gender-segregated teaching in schools, which encouraged them to actively participate in class activities, group projects, and other academic pursuits. Each gender confirmed that they prefer to study in segregated classes, despite the fact that their tradition does not prevent them from doing so. Many tertiary students are even astonished to study in a co-educational classroom for the first time in college, which has a detrimental impact on students' efficacy and motivation. The students came to understand that their socio-cultural milieu not only had an adverse effect on their motivation and self-efficacy, but also on their ability to succeed in school. The students' skill level is moderate.

These results show the need to motivate male students, take into account individual differences between students according to their abilities, and take into consideration the influence of cultural factors and circumstances that may affect students' academic performance.

## Limitations

Our study has several limitations, which can be profitably addressed to stimulate further research. First, the current study was conducted at a private university in the Sultanate of Oman's southern region, which has its own traditions and culture. Second, our study is focusing on self-efficacy, academic achievement, and socio-cultural dimensions. Moreover, the sample of the current study constitutes EFL students studying at the tertiary level in a university with diverse linguistic and cultural backgrounds. Most of the learners were from a mono-gender learning background at school. One of the limitations of the current study is that the teachers are from different cultures; some of them are from Arab countries and some are from western countries, and this may be one of the factors that needs to be studied for its impact on students in future studies. The current study was also conducted during the spread of the COVID-19 pandemic, and the accompanying precautionary measures and the transition from traditional education to distance education.

## Scientific implications

Future studies can focus on EFL learners studying at different levels in universities. Different psychological variables like self-efficacy, self-concept, learner autonomy, self-regulated learning, psychological wellbeing, and social variables like social intelligence, social skills, and empathy can be investigated concerning the mixed-gender learning environment. The current study focused on the learners from a single-gender classroom environment in schools. However, future research can target learners who study in mixed-gender classrooms in schools. Moreover, future studies can focus on how the self-efficacy can be developed by male students in the university context. A study should be conducted on designing a program to enhance self-efficacy among male university students in the Sultanate of Oman, as self-efficacy and Grade Point Average (GPA) of male students are lower than those of female students.

## Data availability statement

The raw data supporting the conclusions of this article will be made available by the authors, without undue reservation.

## Ethics statement

This study was reviewed and approved by Dhofar University. Written informed consent was obtained from all participants for their participation in this study. The treatment of the research participants was fully compliant with the ethical principles set out in the World Medical Association *Declaration of Helsinki* of 1975, as revised in 2008.

## Author contributions

All authors listed have made a substantial, direct, and intellectual contribution to the work and approved it for publication.

## Conflict of interest

The authors declare that the research was conducted in the absence of any commercial or financial relationships that could be construed as a potential conflict of interest.

## Publisher's note

All claims expressed in this article are solely those of the authors and do not necessarily represent those of their affiliated organizations, or those of the publisher, the editors and the reviewers. Any product that may be evaluated in this article, or claim that may be made by its manufacturer, is not guaranteed or endorsed by the publisher.
